# Unique Bisphenol A Transcriptome in Prostate Cancer: Novel Effects on ERβ Expression That Correspond to Androgen Receptor Mutation Status

**DOI:** 10.1289/ehp.10283

**Published:** 2007-08-23

**Authors:** Janet K. Hess-Wilson, Siobhan L. Webb, Hannah K. Daly, Yuet-Kin Leung, Joanne Boldison, Clay E.S. Comstock, Maureen A. Sartor, Shuk-Mei Ho, Karen E. Knudsen

**Affiliations:** 1 Department of Cell and Cancer Biology; 2 Department of Environmental Health; 3 Center for Environmental Genetics and; 4 UC Barrett Cancer Center; 5 University of Cincinnati College of Medicine, Cincinnati, Ohio, USA

**Keywords:** androgen receptor, endocrine disruptor, microarray, prostatic adenocarcinoma, xeno-estrogen

## Abstract

**Background:**

Prostatic adenocarcinomas are dependent on androgen receptor (AR) activity for growth and progression, and therapy for disseminated disease depends on ablation of AR activity. Recurrent tumors ultimately arise wherein AR has been re-activated. One mechanism of AR restoration is via somatic mutation, wherein cells containing mutant receptors become susceptible to activation by alternative ligands, including bisphenol A (BPA). In tumors with specific AR mutations, BPA promotes therapeutic bypass, suggesting significant negative impact to the clinical management of prostate cancer.

**Objective:**

Our goal was to determine the mechanism of BPA action in cancer cells carrying BPA-responsive AR mutants.

**Methods:**

The molecular signature of BPA activity in prostate cancer cells harboring mutant AR was delineated via genetic microarray analysis. Specificity of BPA action was assessed by comparison with the molecular signature elicited by dihydrotestosterone (DHT).

**Results:**

BPA and DHT elicited distinct transcriptional signatures in prostate cancer cells expressing the BPA-responsive mutant AR-T877A. BPA dramatically attenuated estrogen receptor beta (ERβ) expression; this finding was specific to prostate tumor cells in which BPA induces cellular proliferation.

**Conclusions:**

BPA induces a distinct gene expression signature in prostate cancer cells expressing somatic AR mutation, and a major molecular consequence of BPA action is down-regulation of ERβ. Since ERβ functions to antagonize AR function and AR-dependent proliferation, these findings reveal a novel mechanism by which BPA likely regulates cellular proliferation. Future investigation directed at dissecting the importance of ERβ in the proliferative response to BPA will establish the contribution of this event to adverse effects associated with human exposure.

Accruing evidence indicates that exposure to environmental compounds that affect the function of the endocrine system may adversely impact human health. These substances deemed “endocrine disrupting compounds,” or EDCs, are agents that disrupt or enhance known regulatory functions of the endocrine system and function through multiple mechanisms, including alteration of hormone receptor function (Henley 2006; Welshons et al. 2003). While mechanisms by which these effects occur and the level of risk posed to humans have yet to be fully elucidated, the biological significance of EDC exposure can be significant. In humans, a putative link has been established between increased abundance of estrogenic EDCs in the environment and both rising hormone-dependent cancer incidence and reduced fertility (Huff et al. 1996). Thus, recent investigations have placed particular emphasis on delineating the consequence of estrogenic EDC exposure on reproductive tissues.

One such agent, 4,4′-isopropylidene-2-diphenol (bisphenol A, BPA), has been identified as a mitogen for a subset of prostate cancers (Wetherill et al. 2002, 2005, 2006). BPA is a nonplaner plasticizer, which is leached in microgram quantities from polycarbonate plastics and epoxy resins into food and water supplies (Welshons et al. 2003). More than 800 million kilograms of this compound are generated annually in the United States, and up to 95% of adults in the United States have detectable BPA in their urine (Calafat et al. 2005), with adult serum concentrations reported to range in nanomolar concentrations [reviewed by Welshons et al. (2006)]. BPA is known to harbor estrogenic activity; this compound is a weak agonist of both estrogen receptor alpha and beta (ERα and ERβ, respectively) (Kuiper et al. 1997) and is capable of stimulating moderate estrogen-independent proliferation in breast cancer cells (Hess-Wilson et al. 2006; Olsen et al. 2003). The ERs are members of the steroid hormone nuclear receptor family of transcription factors, which are activated by specific steroid hormones and control numerous molecular pathways in hormone responsive tissues, including differentiation and proliferation (Ascenzi et al. 2006; Shang and Brown 2002). Although these reports highlighted a potentially influential role of BPA on ER activity in breast cancer, recent studies reveal BPA as an agonist for mutant androgen receptor (AR) activity in recurrent prostate cancer (Wetherill et al. 2002, 2005, 2006).

Prostatic adenocarcinomas are uniquely dependent on AR activity for growth and proliferation (Trapman and Brinkmann 1996). In prostate cancer cells, androgen [testosterone or dihydrotestosterone (DHT) binding activates the receptor to bind DNA at androgen-responsive elements (AREs), recruit co-activators, and initiate a program of gene transcription that induces cellular proliferation (Lee et al. 1995). Given the reliance of prostate cancer cells on this signal, nullification of AR activity is the first line of therapeutic intervention for disseminated disease, as achieved through either ligand depletion (androgen ablation strategies) or through the use of direct AR antagonists that prevent formation of active AR transcriptional complexes (Klotz 2000; Reid et al. 1999). Although these strategies are initially effective at inducing cell death or cell cycle arrest, recurrent tumors arise within a median of 2–3 years, wherein AR activity has been restored (Feldman and Feldman 2001). One mechanism of AR reactivation is somatic mutation of the AR (estimated to occur in 8–25% of recurrent tumors), where specific mutations of the ligand binding domain render the receptor responsive to an expanded host of ligands (Suzuki et al. 1993; Veldscholte et al. 1990, 1992a, 1992b; Zhao et al. 2000). Remarkably, it has been shown that one of the most common tumor-derived mutant forms of the AR, AR-T877A, is responsive to activation by BPA (Wetherill et al. 2002, 2005). Specifically, it was shown that at environmentally relevant levels of BPA (1 nM), this agent is capable of binding and activating the mutant receptor to induce endogenous expression of *PSA* (prostate specific antigen, a known direct AR target), which is used clinically to monitor prostate cancer development and progression. In cancer cells that express this mutant, low-level BPA exposure induced androgen-independent cellular proliferation, thereby indicating that in the context of AR-T877A, BPA exposure could potentially reduce therapeutic efficacy. This concept was recently validated *in vivo*, wherein BPA accelerated tumor growth after androgen ablation and significantly reduced PSA doubling times (Wetherill et al. 2006). Thus, there is significant evidence that BPA can bolster AR-T877A activity and as a result potentially alter the course of therapeutic response in prostate cancer.

Given the potent effects of BPA on mutant AR activation and prostate cancer progression, it is imperative to determine the molecular underpinning of BPA action. There are differences in prostatic cellular response to BPA compared with DHT, including magnitude of proliferative response, distinctions in ligand binding, and different ligand-induced kinetics of AR recruitment to gene regulatory regions (Wetherill et al. 2002, 2005). Moreover, it is known that differential ligands dictate target gene specificity with regard to the nuclear receptors. Although substantial *in vitro* analyses have revealed the direct binding to and activation of mutant ARs by BPA, it is also well documented that BPA can activate ERα and ERβ, which are expressed in several prostate cancer cell lines [e.g., LNCaP (human prostatic adenocarcinoma cell line)]. However, the BPA-induced cellular proliferation is dependent on AR activation, as blocking of AR function (using the specific AR antagonist, Casodex) reversed all effects of BPA on tumor cell proliferation (Wetherill et al. 2002). Therefore, it is believed that the adverse proliferative effect of BPA is directly through the AR. In our present study, only the proliferation-inducing dose of BPA (1 nM) was used for analysis.

We show by gene expression analyses that low-level, physiologically relevant and proliferation-inducing doses of BPA and DHT elicit overlapping but distinct transcriptional effects in prostate cancer cells expressing the AR-T877A mutation. These data are consistent with previous reports which demonstrate that distinct AR ligands and modes of AR activation alter the specificity of the target genes modified (Davis et al. 2003; Matias et al. 2000; Wilson et al. 1993), and suggest that BPA likely uses mechanisms distinct from DHT to promote cancer cell proliferation. Although these gene-expression differences are complex, interesting and suggestive distinctions were observed. The analyses revealed that BPA exposure in cells expressing AR-T877A triggers a dramatic reduction in expression of ERβ, a nuclear receptor suspected to negatively regulate both AR activity and prostate cancer cell proliferation. Modest reductions in ERβ were observed after DHT treatment. Strikingly, the ability of BPA to modulate ERβ showed cell-type specificity, as alteration in ERβ was observed only after DHT (but not BPA) exposure in cells expressing wild-type AR or the AR-H874Y mutant. Together, these data identify ERβ as a candidate effector of BPA action in prostate cancer cells expressing the AR-T877A mutant and demonstrate that BPA induces a unique transcriptional signature in prostate cancer cells that may be influenced by AR status.

## Materials and Methods

### Reagents

DHT and BPA were purchased from Sigma-Aldrich Chemical Company (St. Louis, MO). Both reagents were solubilized in 100% ethanol to 10^−2^ M and stored at −20°C.

### Cell culture

LNCaP cells were obtained from American Type Culture Collection (Rockville, MD) and were used between passages 28 and 40, maintained in Iscove’s modified Eagle’s medium (Cellgro; Mediatech, Herndon, VA) containing 5% heat-inactivated fetal bovine serum (FBS; Biofluids, Rockville, MD). The 22Rv1 cell line was the gift of J. Jacobberger (Case Western Reserve University, Cleveland, OH) and maintained in Dulbecco’s modified Eagle’s medium containing 10% heat-inactivated FBS. LAPC4 cells were the gift of C. Sawyers (University of California, Los Angeles, CA) and were maintained in Iscove’s modified Dulbecco’s medium (Cellgro; Mediatech) containing 10% heat-inactivated FBS. Media for all cell types were supplemented with 100 U/mL penicillin–streptomycin and 2 mmole/L l-glutamine (Mediatech). Cells were grown in a 5% CO_2_-humidified incubator at 37°C. For culture in steroid-free conditions, cells were seeded in phenol red-free media containing charcoal-/dextran-treated FBS (CDT; Hyclone Laboratories, Logan, UT).

### Bromodeoxyuridine incorporation assay

Cells were seeded in six-well dishes on poly-l-lysine–coated coverslips at a density of 2.5 × 10^5^ cells per well into CDT media (allowing for depletion of AR ligands and AR activity), then supplemented with either vehicle (0.1% ethanol), 0.1 nM DHT, or 1 nM BPA the following day. After 24 hr of treatment, cells were labeled with cell proliferation-labeling reagent (Cell Proliferation Labeling reagent; Amersham, Buckinghamshire, UK) according to manufacturer’s protocol, for 18 hr. Cells were then processed to detect bromodeoxyuridine (BrdU) via indirect immunofluorescence. Experiments were performed with at least six independent biological replicates, and at least 250 cells per experiment were tallied per replicate for each condition. Averages and standard deviation are shown.

### Microarray hybridization

Vehicle control (0.1% EtOH), 0.1 nM DHT or 1 nM BPA were added to LNCaP cells 24 hr after seeding into CDT media. For each condition, three independent samples were generated for microarray analyses. After 24 hr of exposure to reagents, total RNA was isolated via TRIzol extraction for microarray analysis from each independent biological replicate (*n* = 3 per condition). The microarray experiments were carried out essentially as described in published reports and references therein (Guo et al. 2004; Sartor et al. 2004). The human 70-mer oligonucleotide library version 2 (22,291 optimized oligos) (QIAGEN, Alameda, CA) was suspended in 3× SSC (sodium chloride–sodium citrate) at 30 μM and printed at 22°C and 65% relative humidity on aminosilane-coated slides (Cel Associates, Inc. Pearland, TX) using a high-speed robotic Omnigrid machine (GeneMachines, San Carlos, CA) with Stealth SMP3 pins (Telechem, Sunnyvale, CA).

Fluorescence-labeled cDNAs were synthesized from total RNA using an indirect amino allyl–labeling method via an oligo(dT)-primed, reverse transcriptase reaction. Imaging and data generation were carried out using a GenePix 4000A and GenePix 4000B (Axon Instruments, Union City, CA) and associated software from Axon Instruments (Foster City, CA). The microarray slides were scanned with dual lasers with wavelength frequencies to excite cyanine (Cy) 3 and Cy5 fluorescence emittance. Images were captured in JPEG and TIFF files, and DNA spots captured by the adaptive circle segmentation method. Information extraction for a given spot is based on the median value for the signal pixels minus the median value for the background pixels to produce a gene set data file for all the DNA spots. The Cy3 and Cy5 fluorescence signal intensities were normalized.

### Statistical analysis and functional testing of microarrays

The data representing background subtracted spot intensities generated by GenePix Pro software ,version 5.0 (Axon Instruments) were analyzed to identify differentially expressed genes. Data normalization was performed in two steps for each microarray separately (Guo et al. 2004; Sartor et al. 2004). First, background adjusted intensities were log-transformed and the differences (*M*) and averages (*A*) of log-transformed values were calculated as *M* = log_2_(×1) − log_2_(×2) and *A* = [log_2_(×1) + log_2_(×2)]/2, where ×1 and ×2 denote the Cy5 and Cy3 intensities, respectively. Second, normalization was performed by fitting the array-specific local regression model of *M* as a function of *A*. Normalized log-intensities for the two channels were then calculated by adding half the normalized ratio to *A* for the Cy5 channel and subtracting half the normalized ratio from *A* for the Cy3 channel. The statistical analysis was performed for each gene separately by fitting the following analysis of variance (ANOVA) model (Dudiot and Fridlyand 2002). Resulting *t*-statistics from each contrast were modified using an empirical Bayesian moderated-T method (Smyth 2004). This method uses variance estimates from all genes to improve the variance estimates of each individual gene. Estimates of fold change were calculated, and genes with *p*-value < 0.05, fold change > 2, and average spot intensity > 100 were considered for follow-up. Data analysis was performed using the statistical software R (http://www.bioconductor.org) and the Bioconductor platform (http://www.r-project.org). Average linkage hierarchial clustering was performed using the uncentered correlation similarity metric for the genes considered for follow-up.

The gene list was analyzed to determine which gene categories were enriched with differentially expressed genes using EASE/ DAVID (Hosack et al. 2003), and the gene sets tested were the three branches of the Gene Ontology (GO) database (http://www.geneontology.org). Fisher’s exact probability, using the FDR (false discovery rate) multiple testing adjustment, was calculated for each GO term (Hosack et al. 2003).

### Reverse transcriptase polymerase chain reaction (PCR)

RNA was isolated from cells by TRIzol extraction, of which 5 μg was used to generate cDNA with random hexamers using the ThermoScript RT-PCR system (Invitrogen, Carlsbad, CA). PCRs were performed using the following primer sets: *PSA* forward: CTTGTAGCCTCTCGTG GCAC; *PSA* reverse: GACCTTCATAG CATCCGT GAG; *GAPDH* forward: CCACCCCATG GCAAATTCCATGCA; *GAPDH* reverse: TCTAGACGGCAGGTC AGGTCCACC; *ER*β forward: CAGCAT TCCCAGCAAT GTCAC; *ER*β reverse: GGTAAGGT GTTCTAGCGATCTTG; *WISP3* forward: AAGCAGGCTCTGG GCAGCTA; *WISP3* reverse: GACGTTGT TGCAGTTCC; *FKBP5* forward: GAGAAA AGCCAGCATAAAGC; *FKBP5* reverse: TCTAGAACTTGCGTGGAAAG. Amplifications were performed using Taq DNA polymerase with the following conditions: PSA and *GAPDH* 94°C 30 sec, 54°C 30 sec, and 72°C 30 sec; *ER*β 94°C 30 sec, 56°C 1 min, and 73°C 1 min; *WISP3* 95°C 30 sec, 60°C 30 sec, and 68°C 30 sec; *FKBP5* 95°C 30 sec, 56°C 1 min, and 73°C 1 min. Resulting products were resolved by agarose gel electrophoresis.

### Real-time PCR analysis of ERβ

Five micrograms of total RNA were reverse-transcribed into cDNA by Superscript III reverse transcriptase (Invitrogen) according to the manufacturer’s instructions. Real-time PCR reactions containing Power SYBR Green PCR Master Mix (Applied Biosystems), 250 ng cDNA template and 500 nM of ERβ specific primers (ERβ forward: 5′-TGG CTA ACC TCC TGA TGC TC-3′; ERβ reverse: 5′-TCC AGC AGC AGG TCA TAC AC-3′) were prepared. Real-time PCR reactions in an Applied Biosystems Prism 7900HT (Applied Biosystems) were initiated by heating to 50°C for 2 min and then to 95°C for 10 min. They were followed by 40 cycles of denaturation (95°C for 15 sec) and annealing/extension (63°C for 30 sec). Dissociation curves of each reaction were collected to evaluate the quality of the end products. A standard curve for ERβ was constructed by serial dilutions of the expression vector prepared previously (Leung et al. 2006). The copy number was determined according to the published formula provided in the Applied Biosystems manual (http://www.appliedbiosystems.com/). Loading control was normalized by using *hGAPDH* level using a published method (Leung et al. 2006). Statistically analysis was performed using one-way ANOVA with Newman–Keuls test for multiple comparisons: *p < 0.05, **p < 0.01.

### Immunoblotting

Cells were treated as described for RNA isolation. However, for protein analyses, total cells were pelleted and whole cell lysates prepared using r adioimmunoprecipitation buffer supplemented with standard protease inhibitors and phenyl-methylsulfonyl fluoride. Lysates were subjected to brief sonication and clarified by centrifugation. Equal protein concentrations (∼ 20 μg) were loaded and subjected to sodium dodecyl sulfate–polyacrylamide gel electrophoresis. Proteins were transferred to Immobilon membrane (Millipore Corp., Bedford, MA) and immunoblotted for ERβ or CDK4 (cyclin dependent kinase 4, loading control) (antisera: H-150 and H-22, respectively; Santa Cruz Biotechnology, Santa Cruz, CA). Goat-anti rabbit (Alexa Fluor 680 A21076 1:10,000; Molecular Probes, Eugene, OR) was used to visualize the antibody–antigen complex.

## Results

### BPA initiates androgen-independent prostate cancer cell proliferation in cells harboring the AR-T877A mutation

**Figure 1:**
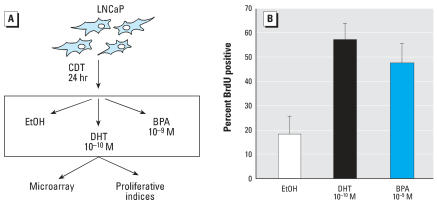
BPA induces androgen-independent cellular proliferation in cells expressing AR-T877A. (*A*) LNCaP cells were cultured under conditions of steroid hormone ablation (5% charcoal-/dextran-treated serum, CDT) and subsequently stimulated with either DHT, BPA, or vehicle control [0.1% ethanol (EtOH)]. (*B*) Cells cultured as in *A* were pulse labeled with BrdU for the last 16 hr of treatment, fixed, and BrdU incorporation was quantified by indirect immunofluorescence. Percent BrdU positive cells is shown. Error bars represent mean ± SD.

To dissect the mechanisms by which BPA modulated mutant AR activity, conditions were used wherein BPA exposure resulted in a significant proliferative advantage (Figure 1A). Briefly, AR-T877A–expressing prostate cancer cells (LNCaP) were initially cultured in hormone-depleted conditions (CDT), to suppress cell cycle progression and deplete endogenous AR of ligand. After 24 hr, cells were treated with DHT, BPA, or vehicle (0.1% ethanol; EtOH) and pulsed with 5-bromo-2-deoxyuridine (BrdU) to measure G_1_-S phase progression. As expected, androgen depletion significantly attenuated cell cycle progression compared with cells cultured in the presence of physiological levels of DHT (0.1 nM) (approximate 3-fold reduction in BrdU incorporation, Figure 1B). Consistent with previous reports for cells carrying BPA-responsive AR mutants (Wetherill et al. 2002, 2005), BPA exposure also induced significant S-phase progression compared with vehicle control (∼ 2.5-fold induction over vehicle control). Similar trends were observed after increasing time periods postligand stimulation or when AR ligand stimulation was applied after prolonged periods of hormone ablation (data not shown). Previous studies showed that BPA administration results in dose-dependent proliferation (with concomitant increase in cell number) of LNCaP cells in the absence of androgen (Wetherill et al. 2002, 2005, 2006). Therefore, in this experimental paradigm, DHT and BPA each elicit significant effects on cellular proliferation, and these strategies can be used to dissect the molecular impact of BPA on prostate cancer.

### BPA elicits a distinct transcriptome in prostate cancer cells

**Figure 2:**
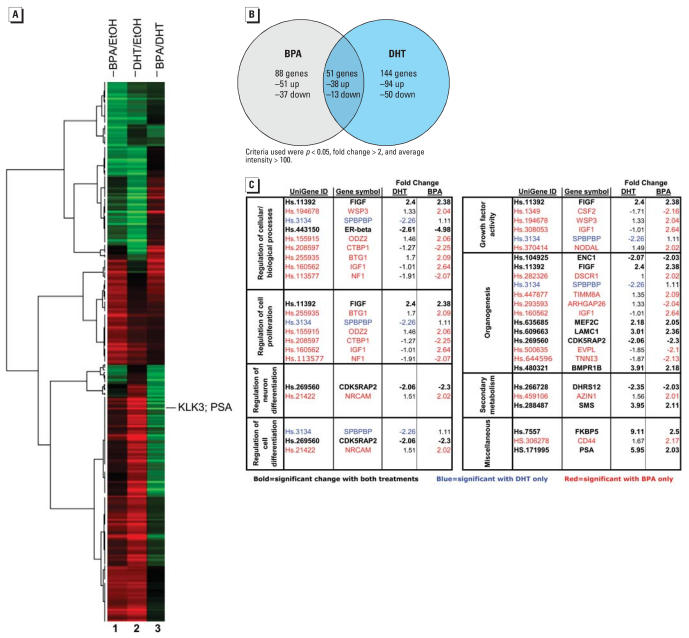
BPA induces a unique transcriptome in prostate cancer cells expressing AR-T877A. Microarray analyses were performed using triplicate biological replicates (*n* = 9) and statistical analyses performed as described in “Materials and Methods.” (*A*) Heat map of the fold change estimates, averaged across sample, showing all genes with a statistically significant, > 2-fold alteration over vehicle control. Column 1 is statistically significant changes in averaged gene expression in BPA-treated samples compared with EtOH control; column 2 represents the statistically significant changes in expression after DHT relative to EtOH control. Column 3 is a comparative analysis and represents the genes that were statistically significant for BPA exposure compared with DHT treatments. Green bars indicate reduced expression, and red bars indicate induced gene expression. The bar representing PSA (*KLK3*) is indicated. Microarray experiment was performed on three independent experiments in triplicate. (*B*) VENN diagrams highlight the disparity in DHT and BPA effects on gene regulation. *C*) Gene Ontology was performed as described in “Materials and Methods.” Categories passing statistical significance are shown. Gene annotations are from Unigene (http://www.ncbi.nlm.nih.gov/sites/entrez?db=unigene).

To determine the specific effects of BPA that underlie its pro-proliferative capacity, parallel cultures were examined for alterations in gene expression. Briefly, cells were treated as described in Figure 1 with either vehicle (EtOH), DHT, or BPA before RNA isolation. A time course was initially performed, where it was determined that BPA-induced PSA expression peaks at 24 hr poststimulation compared with approximately 16 hr after DHT stimulation (data not shown). The delay in peak observation is consistent with previous observations that AR nuclear translocation facilitated by BPA is delayed compared with DHT-induced AR translocation (Wetherill et al. 2002). Therefore, this time point was chosen for subsequent microarray analyses. RNA for each condition (EtOH, DHT, and BPA) was isolated from three independent experimental cultures to determine the change in transcript levels for each condition and when evaluated in triplicate, genes passing the ANOVA criteria were further analyzed to create a cluster heat map. A heat map was generated using the fold change estimates and averaged across samples. As can be seen in Figure 2A, BPA (column 1) and DHT (column 2) showed some similar gene expression profiles with comparable patterns of gene down-regulation (green) and up-regulation (red) relative to the vehicle control. However, column 3 provides a visual mechanism to compare the profile of BPA with that of DHT and exemplifies the differences in the genetic profile induced by the two ligands. An important control and point of reference, *KLK3* (prostate-specific antigen, *PSA*, UniGene ID# Hs.171995) was up-regulated by both BPA and DHT but was more robustly activated by DHT, as can be seen by the green band in lane 3. Statistical analyses revealed that a total of 283 genes showed significant alteration relative to vehicle control (with *p* < 0.05, > 2-fold change in gene expression and average intensity > 100 as criteria for significant changes). Of these, only 51 genes were common to both BPA and DHT (38 up-regulated and 13 down-regulated). However, 88 genes were uniquely regulated by BPA treatment (51 up-regulated and 37 down-regulated), and 144 genes were exclusive to DHT treatment (94 up-regulated and 50 down-regulated) (Figure 2B). These data suggest that although the proliferative response to DHT or BPA is congruent, the gene profiles after exposure are divergent between the canonical ligand DHT and the environmental contaminant BPA.

Key target genes critical for DHT-mediated AR-dependent proliferation are largely unknown. Therefore, the expression changes induced by mitogenic BPA or DHT were also analyzed via GO to determine cellular pathways that may be altered by AR activation with these disparate ligands (Figure 2C). The GO terms that were affected are of significant interest to prostate development and organ homeostasis and function, as well as prostatic disease, and include genes involved in the regulation of cell proliferation, differentiation, organogenesis, and growth factor pathways. Among these categories, several previously described direct or suspected AR gene targets were identified in both the DHT- and BPA-treated cohorts, including prostate specific antigen (*KLK3*/ PSA), and *FKBP5* (FK506 binding protein 5; UniGene ID Hs.7557), a member of the immunophilin protein family involved in protein trafficking and folding. Notably, *FKBP5* has been shown to be up-regulated in xenograft models of androgen-independent prostate cancer and induced by androgen exposure in LNCaP cells (Magee et al. 2006). In addition, several potentially key effectors of prostate cancer cell proliferation were identified in the BPA-treated cohort, including *FIGF* (c-fos–induced growth factor, also called VEGF-D; UniGene ID Hs.11392), which has been associated with advanced-stage metastatic prostate cancer (Kaushal et al. 2005). The *WISP3* (WNT-1 inducible signaling pathway protein 3, UniGene ID Hs.194678) gene, whose protein product is a member of the CCN (connective tissue growth factor, cysteine-rich protein 61, and nephroblastoma overexpressed gene) family, was also among those induced by BPA. *WISP3* is thought to have important roles in carcinogenesis and has identified functions in cell growth and differentiation (Kleer et al. 2004). Other potential effectors of BPA action included *SPBPBP* (DNA-binding protein amplifying expression of surfactant protein B; UniGene ID Hs.3134), *CRBP1*, [cellular retinol-binding protein type 1; UniGene ID Hs.208597, a member of the retinoids, known to be involved in cell growth, differentiation, and carcinogenesis, and which specifically has been found to have altered methylation patterns in prostate cancer (Jeronimo et al. 2004; Suzuki et al. 2006)], and *IGF1* [insulin-like growth factor (UniGene ID Hs.160562), thought to induce cell survival/proliferation and a known activator of ligand-independent AR activity (Craft et al. 1999; Culig et al. 1994)]. Last, the most significantly altered gene in the BPA data set was *ER*β (UniGene ID# Hs.443150), which has been proposed to antagonize AR activity and limit proliferation in prostatic epithelia (Imamov et al. 2004a, 2004b; Weihua et al. 2002).

**Figure 3:**
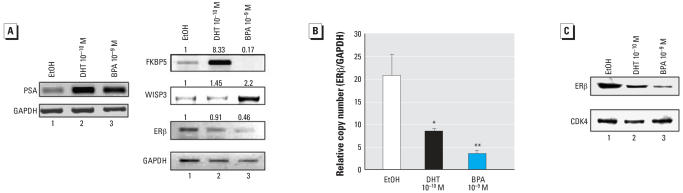
Validation of selected targets reveals that BPA significantly down-regulates ERβ expression in cells expressing the BPA-responsive AR-T877A mutant. *A*) Validation of alterations in PSA expression was performed by RT-PCR. As expected, both DHT and BPA induced PSA expression in cells expressing AR-T877A (left panel). In agreement with the microarray, it was also noted that BPA induces down-regulation of ERβ. By contrast, marked induction of WISP3 was also noted, and no effect was observed with FKBP5 (right panel). Numbers correspond to the band intensity of each sample set to EtOH (EtOH = 1) and relative to the GAPDH RNA control. *B*) The impact of BPA on ERβ was quantified by real-time PCR, and relative copy number was determined. Error bars represent mean ± SD; *n* = 9; *p < 0.05, **p < 0.01. (*C*) Representative (*n* = 3) immunoblot of ERβ protein levels after BPA or DHT exposure. As shown, BPA exposure causes a marked reduction in ERβ accumulation.

Given the potential importance of these factors in facilitating BPA-induced cellular proliferation, several of the pro-proliferative genes and/or AR target genes were analyzed for alteration in response to BPA treatment. Using conditions identical to those used for the microarray studies, cells were placed in an environment of androgen ablation and subsequently stimulated with DHT or BPA for 24 hr. Initially, the AR target gene PSA was analyzed. As expected, both DHT and BPA increased PSA mRNA expression (Figure 3A, left panel). Conversely, FKBP5 was increased by DHT (8-fold over EtOH) but not significantly induced by BPA treatment (Figure 3A, right panel). By contrast, BPA treatment increased WISP3 (2-fold over EtOH control), whereas DHT had no significant effect on gene expression (Figure 3A, right panel). Last, analyses of ERβ transcript levels were explored by reverse transcriptase PCR, as reduction of this mRNA was initiated by both DHT and BPA (compare –2.6 fold change by DHT with –4.8-fold change by BPA in Figure 2C). Interestingly, both microarray and reverse transcriptase PCR validation showed that BPA facilitated more significant down-regulation of ERβ than DHT (Figure 3A, right panel, lane 3; 0.46-fold expression compared with EtOH for BPA).

Real-time PCR was used to accurately quantify the level of ERβ transcript down-regulation induced by DHT and BPA treatment of LNCaP prostate cancer cells. As shown in Figure 3B, cells cultured under conditions of androgen ablation (EtOH vehicle treatment) demonstrated approximately 20 relative copy numbers of ERβ transcript. DHT treatment reduced ERβ transcript to < 10 (a reduction of 50%, *p* < 0.05). Remarkably, BPA exposure dramatically decreased ERβ transcript copy number (∼ 4, 80% reduction, *p* < 0.01; Figure 3B). There was not a strong statistical difference between the DHT- and BPA-mediated reduction in ERβ (*p* > 0.05). These results were further validated at the protein level in parallel assays by immunoblotting for ERβ. Therein, exposure of the cells to mitogenic doses of BPA and DHT resulted in a detectable decrease in ERβ protein (Figure 3C, compare lanes 1, 3). CDK4 was used as the loading control. Collectively, these data indicate that although both BPA and DHT can activate mutant AR and resultant cellular proliferation in prostate cancer cells, each agent induces a unique molecular signature, wherein genetic profiles induced by mitogenic doses of DHT or BPA showed both overlapping (e.g., PSA and ERβ) and divergent (e.g., FKBP5, WISP3) responses.

### BPA-mediated regulation of ERβ is cell-type specific

**Figure 4:**
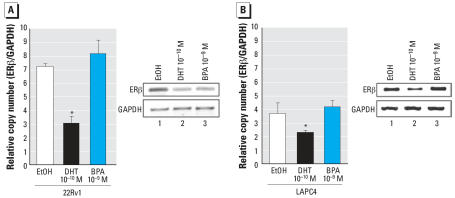
Specificity of BPA-mediated modulation of ERβ. ERβ expression was monitored using at least two independent biological replicates for each condition and cell line, analyzed in triplicate and quantified by real-time PCR in cells expressing AR-H874Y (22Rv1, *A*) or wild-type AR (LAPC4, *B*). Error bars represent mean ± SD.

The observation that BPA significantly reduced ERβ expression was striking, as this receptor plays significant roles in both prostate development and disease progression (Adams et al. 2002; Leav et al. 2001). Therefore, the specificity of BPA-induced ERβ down-regulation was determined. The effect of BPA treatment on ERβ transcript levels was examined in two additional prostate cancer cell lines, 22Rv1 and LAPC4. 22Rv1 cells are androgen-independent prostate cancer cells that express the AR-H874Y mutant. 22Rv1 cells were treated under identical conditions as previously described, cells were switched to steroid deprived media for 24 hr, then treatments (EtOH vehicle control, DHT or BPA) were added for 24 hr. As shown, cells treated under conditions of hormone ablation demonstrated a relative copy number of 7 (ERβ over GAPDH control), as determined by quantitative reverse transcriptase PCR. DHT reduced the level of ERβ transcript copy by approximately 50% (*p* < 0.05); however, contrary to observations in LNCaP cells, exposure to BPA had no effect on ERβ transcript copy number (Figure 4A).

The impact of BPA exposure on ERβ transcript was examined further in the LAPC4 prostate cancer cell line, which harbors wild-type AR and is androgen dependent. Strikingly, although the relative level of ERβ was lower in LAPC4 cells, DHT treatment still resulted in approximately a 40% reduction in ERβ gene transcript (*p* < 0.05). However, similar to the effect seen in 22Rv1 cells, BPA exposure did not alter ERβ transcript levels (Figure 4B), as evidenced by further reverse transcriptase PCR (right panel) and quantified by quantitative PCR (left panel). Combined, these data demonstrate that the effect of BPA on reduction of ERβ gene expression is cell specific and correlated with cells expressing the BPA-responsive AR-T877A mutant.

## Discussion

Somatic mutation of the AR is known to drive recurrent tumor formation in a significant percentage of patients undergoing hormone therapy (Feldman and Feldman 2001; Hirawat et al. 2003; Marcelli et al. 2000; Navarro et al. 2002; Taplin et al. 2003; Veldscholte et al. 1992a), and it has been shown previously that selected AR mutants become receptive to activation by the environmental contaminant BPA. The clinical ramifications of BPA activating tumor-derived mutant ARs and inducing androgen-independent tumor cell proliferation may be substantial, as BPA can reduce therapeutic efficacy in xenograft models (Wetherill et al. 2006). While these data point toward the potential for BPA to assist tumor cells in escaping therapy, the molecular mechanisms of this process were not well understood. In this study, the molecular consequence of BPA action was identified under conditions in which BPA promotes androgen-independent proliferation. Gene expression analyses revealed that in AR-T877A–expressing cells, BPA elicited an overlapping but distinct molecular signature compared with that induced by exposure to canonical AR ligand (DHT). Unexpectedly, detailed examination of the most significant targets demonstrated that BPA exposure elicited dramatic reduction in expression of ERβ, a nuclear receptor that is proposed to antagonize AR activity and androgen-dependent proliferation in prostatic epithelia (Leav et al. 2001; Pravettoni et al. 2007; Weihua et al. 2001). Comparative analyses revealed that while DHT exposure also reduced ERβ, this effect was marginal compared with the BPA response. Last, the ability of BPA (but not DHT) to attenuate ERβ function appears to be specific to cell type and putatively receptor specific, as BPA had no detectable effect on ERβ expression in cells containing wild-type AR or AR-H874Y. Notably, these also represent cell types in which BPA fails to induce cellular proliferation. Together, these data indicate that the ability of BPA to induce androgen-independent cellular proliferation is associated with AR-T877A expression and a unique gene expression signature that involves down-regulation of ERβ.

Combined with these observations, the concept that BPA induces cell-specific transcriptional profiles is emerging. For example, transcriptional analysis of BPA exposure on breast cancer cells (MCF-7) revealed that BPA up-regulated a significant number of genes involved in cell cycle progression and purine and pyrimidine metabolism (Shioda et al. 2006), reaffirming the proliferative biological action of this compound in estrogen-dependent tissues. Interestingly, it has been shown that BPA exposure increases ERβ in some breast cancer lines (Cappelletti et al. 2003), suggesting that the direct activation of ERs by BPA may facilitate the proliferative response to these tissues. Additionally, it has been shown that higher levels of BPA uniquely regulate growth- and development-related genes in breast cancer cells (Singleton et al. 2006). From these data it is clear that in estrogen-sensitive mammary tissues, BPA exposure modulates pathways with important biological outcomes (e.g., cell cycle progression), and that the effects of BPA augment the known proliferative response to ER activation in this tissue type.

The ability of BPA to induce ERβ expression is in marked contrast to the observations herein, where BPA significantly reduced ERβ mRNA and protein accumulation. Although the biological functions of ERβ remain poorly understood, in contrast to the proliferative role of ERβ in mammary tissue, it is hypothesized that functions of ERβ in the prostate include antiproliferative action and regulation of apoptosis (Lau et al. 2000; Leav et al. 2001; Mak et al. 2006; Pravettoni et al. 2007). Studies have demonstrated that loss of ERβ signaling in prostatic epithelium results in increased proliferation (Horvath et al. 2001; McPherson et al. 2007; Pasquali et al. 2001) and up-regulation of AR (Imamov et al. 2004a). Molecular analyses have shown that ERβ can form a direct complex with AR and abrogate its activity (Migliaccio et al. 2000). Moreover, direct analysis of prostate disease progression has shown that ERβ expression declines as prostate cancer develops [although expression is regained in lymph node and bone metastasis (Horvath et al. 2001; Ji et al. 2005; Lai et al. 2004; Leav et al. 2001)]. In mouse model systems, prostatic epithelial hyperplasia can be attenuated by specific activation of ERβ (Imamov et al. 2004a, 2004b; McPherson et al. 2002; Weihua et al. 2001 Weihua et al. 2007). In the human population, the chemo-prevention and protective nature of phytoestrogens in prostate cancer is hypothesized to rely on the high binding affinity and activating potential of these estrogenic compounds for ERβ (Kuiper et al. 1998; Leung et al. 2006). Collectively, these data indicate that ERβ plays a significant role in regulating normal prostate homeostasis (Horvath et al. 2001; Leav et al. 2001; Weihua et al. 2001), and that loss of ERβ plays a possible role in enhancing the survival of prostate cancer cells (Kim et al. 2002; Lau et al. 2000; Neubauer et al. 2003). The present observation that BPA initiates ERβ down-regulation suggests that this may be one mechanism by which BPA bolsters overall AR activity, thereby promoting a proliferative response. The observation that DHT modestly altered ERβ levels was not expected, as ERβ has not been well documented as a target (direct or indirect) of androgen action. Of note, however, is that a significant fraction of published microarray studies using the AR-dependent prostate cancer cell lines have been performed using high doses of androgen (≥10 nM) (Dehm and Tindall 2006), which induce profound cell cycle arrest in prostate cancer cells. Thus, it is possible that effects on ERβ may be masked by such experimental conditions. Furthermore, scrutiny of the literature demonstrated that down-regulation of ERβ by DHT has been previously observed but had not yet been validated (Arnold et al. 2005; Segawa et al. 2002). While the complete molecular complexities of mitogenic signaling in prostate cancer cells remain to be elucidated, it would be interesting to continue this line of experimentation with an analysis of gene regulation as a function of time with the various mitogenic ligands. However, together these collective observations put forward the intriguing hypothesis that the proliferative effects of BPA may be at least partially due to the manipulation of ERβ levels.

The ability of BPA to govern ERβ levels was observed only in cells expressing the AR-T877A mutant. This observation shows critical specificity in BPA action; cells expressing wild-type AR or AR-T874Y, which are nonresponsive to the mitogenic effects of BPA (Wetherill et al. 2005), were impervious to the BPA-facilitated changes in ERβ. Although presently the possibility that cell-type specificity is controlled by factors in addition to AR mutation, it is intriguing to speculate about the role of mutant AR in facilitating BPA-mediated ERβ regulation. Previous work has shown that BPA can activate AR-H874Y and elicit transactivation of target genes in reporter assays (Wetherill et al. 2005); however, because of the ligand-independent nature of this cell line, AR activation by either DHT or BPA does not facilitate proliferation in AR-H874Y–dependent (22Rv1) cells (Wetherill et al. 2005). With regard to wild-type AR, BPA is not known to bind to or activate this receptor at the doses used, and LAPC4 cells (which express wild-type AR) are not responsive to androgen-independent cellular proliferation by BPA exposure. By comparison, DHT-mediated reduction of ERβ expression was relatively equivalent in each of these lines, thus indicating that the capacity to modulate ERβ remains intact. Hence, the ability of BPA to alter ERβ levels appears to correlate to the mitogenic capacity of BPA. Future studies will be directed at dissecting the mechanism by which AR ligands alternately regulate ERβ and the contribution of differential mutations of AR to this process. In addition, given that BPA can bind to ERβ directly, it will also be important to determine whether the impact of BPA on ERβ is direct or indirect. Recent work by Susiarjo et al. (Susiarjo et al. 2007) have demonstrated that the adverse effects of BPA on oocyte development requires ERβ further supporting the notion that the salient mode of action for BPA may be through alterations in ERβ function. Remarkably, it still remains largely unknown what controls ERβ expression in the prostate. Although epigenetic regulation of ERβ in prostate cancer has been reported (Zhu et al. 2004), knowledge of what *cis*-acting factors directly interact with the ERβ promoter is very limited; AP-2 was recently found to be a promising transcription factor that regulates ERβ gene expression (Zhang et al., in press).

In summary, the present study demonstrates that BPA uses a cell-specific gene expression signature in prostate cancer cells expressing a somatic mutation of AR, and that a major molecular consequence of BPA action is down-regulation of ERβ These studies provide insight into a potentially novel mechanism by which BPA can promote AR-T877A activity and androgen-independent cellular proliferation. Given the critical importance of controlling AR activity for prostate cancer therapy, the present study provides the foundation for future investigations directed at dissecting the role of BPA and ERβ in controlling prostate cancer growth and/or progression.
